# Hedgehog Zoonoses

**DOI:** 10.3201/eid1107.050045

**Published:** 2005-07

**Authors:** Melissa Behr

**Affiliations:** * New York State Department of Health, Albany, New York, USA

**Keywords:** zoonoses, bioterrorism, infection

**To the Editor:** The article on hedgehog zoonoses (1) reviews diseases transmitted from African and European hedgehogs to humans but does not compare their infectious potential to that of other animals and people. For example, cats and Yorkshire terriers are well-known vectors of ringworm (2), but this has not been highlighted in Emerging Infectious Diseases. Also, the reports of herpesvirus (including human herpes simplex) hepatitis described in the article occurred as fatal hepatitis in hedgehogs, whereas their owners apparently escaped unscathed. These cases appear to be "reverse zoonoses" that are dangerous for the pet but not its human contacts. Perhaps the misleading table in the article should be revised so that busy medical doctors don't jump to conclusions, and hedgehogs don't end up on the euthanasia list at shelters.
