# Supplementary material for: Hedgehog Zoonoses
Source: Emerg Infect Dis. 2005 Jul;11(7):1146. doi: 10.3201/eid1107.050045 (PMC3371789; doi:10.3201/eid1107.050045)
Supplement: Related texts

# Reply: Hedgehog Zoonoses

**DOI:** 10.3201/eid1107.050410

**Authors:** Bruno B. Chomel, Patricia Y. Riley

**Affiliations:** * University of California–Davis, Davis, California, USA

**In response:** We thank Dr. Behr for her comment (1). The intent of our manuscript was to report, from a literature review, information on zoonotic infections related to hedgehogs. Of course, we are mainly concerned with infections or infestations that hedgehogs can transmit to humans, but we also noted that the inverse can be true, and humans can be a source of infection in pet hedgehogs. This manuscript was intended to inform not only physicians but also veterinarians and wildlife rescuers who may not be familiar with zoonotic diseases borne by or transmitted to hedgehogs. We also would like to take advantage of this letter to clarify a few points from our manuscript. First of all, pet hedgehogs are mainly African pygmy hedgehogs, and no reliable data are available regarding the number of European hedgehogs that are kept as pets either in Europe, the United States, or other parts of the world. In many European countries, native hedgehogs are protected by law and cannot be kept as pets (F. Moutou, pers. comm.). Furthermore, our comment on plague and "hedgehogs" in Madagascar was meant to be informative, as these animals are found only on that island. They are not true hedgehogs (belonging to the family *Tenrecidae* and not *Erinaceidae*) and are unlikely to be kept as pets (2; F. Moutou, pers. comm.). In our literature review from PubMed, we found no report of human leptospirosis infection from hedgehogs. However, the European hedgehog is considered the main host of *Leptospira bratislava* in the Netherlands and Denmark and the main host of *L. canicola* in Israel (2). Finally, if hedgehogs can be infected by lungworms of the genus *Capillaria*, no report of a human infection transmitted by hedgehogs has been published to our knowledge.

## References

[1] Behr M. Hedgehog zoonoses. Emerg Infect Dis. 2005;11:1146.1603279210.3201/eid1107.050045PMC3371789

[2] Smith JMB. Diseases of hedgehogs. Vet Bull. 1968;38:425–30.
